# Anemia of Chronic Disease: Pathophysiology, Diagnosis and Management

**DOI:** 10.3390/hematolrep18040048

**Published:** 2026-07-02

**Authors:** Keziah Abbotts, Priya Sriskandarajah

**Affiliations:** Department of Haematology, Guy’s Hospital, Great Maze Pond, London SE1 9RT, UK; priya.sriskandarajah@nhs.net

**Keywords:** anemia of chronic disease, inflammation, functional iron deficiency

## Abstract

Anemia of chronic disease (ACD) is a condition linked to chronic immune activation secondary to a wide range of infectious, inflammatory, and autoimmune diseases. It is characterized by a state of iron-restricted erythropoiesis, in which prolonged activation of cytokines leads to retention of iron within the reticulo-endothelial system, driven primarily by hepcidin. Reduced iron availability contributes to a blunted response by erythropoietin and impaired erythropoiesis, in addition to a shortened red cell lifespan. In patients found to have anemia and evidence of chronic inflammation, parameters such as mean cell volume, iron studies, percentage of hypochromic red cells, reticulocyte hemoglobin content, and levels of ferritin, serum transferrin receptor, hepcidin, erythropoietin, and GDF15 are all used to build a picture of anemia of chronic disease. Following this, management normally utilizes erythropoietin-stimulating agents alongside parenteral iron supplementation when treatment of the underlying cause is not available. Newer therapies, such as hypoxia-inducible factor prolyl hydroxylase inhibitors and hepcidin inhibitors, also play a role, while cytokine targets, carbon dots, androgens, and other therapies are emerging as possible treatment routes. Despite its high prevalence, there remain few standardized methods of diagnosis or management in anemia of chronic disease. This narrative review explores long-standing and emerging practices in the diagnosis and management of this condition to ensure an up-to-date understanding.

## 1. Introduction

Anemia is a pathological state in which the rate of red cell production cannot match the rate of red cell destruction, leading to a reduced hemoglobin (Hb) concentration [1]. The World Health Organization defines anemia as a state in which the oxygen-carrying capacity of red cells is inadequate to meet physiological requirements, with hemoglobin levels of <120 g/L in women and <130 g/L commonly used for diagnosis [2].

Anemia of chronic disease (ACD), often termed ‘anemia of inflammation’, arises secondary to chronic immune activation [3]. It is the second most prevalent cause of anemia worldwide after iron deficiency anemia (IDA) and the leading cause of anemia in hospitalized patients [4]. ACD occurs across a broad spectrum of chronic conditions, including autoimmune diseases, chronic infections, malignancies, chronic kidney disease (CKD), heart failure, and inflammatory disorders (Table 1). The underlying pathophysiology of chronic inflammation can lead to reduced iron availability for erythropoiesis [4,5]. Unlike IDA, which results from absolute depletion of iron stores, ACD is characterized by functional iron deficiency. In this setting, iron becomes sequestered within macrophages and hepatocytes, rendering it unavailable for effective erythropoiesis [6,7]. This distinction is challenging but important because diagnostic interpretation and treatment strategies differ substantially.

Although considerable advances have been made in understanding the biology of ACD, no universally accepted diagnostic criteria currently exist [8]. Traditional markers such as ferritin and transferrin saturation remain imperfect in inflammatory states, while newer biomarkers, including serum transferrin receptor (sTfR), reticulocyte hemoglobin content (CHr), hepcidin, and growth differentiation factor-15 (GDF15) continue to be evaluated [9,10,11]. Similarly, treatment approaches vary considerably across disease settings, and international recommendations remain heterogeneous.

The purpose of this narrative review is to provide a clinically focused update on the pathophysiology, diagnosis, and management of ACD. Particular emphasis is placed on practical approaches to distinguishing ACD from IDA, interpretation of emerging diagnostic biomarkers, contemporary management strategies, and the evolving therapeutic landscape.

**Special Considerations in Chronic Kidney Disease**

Although chronic kidney disease-associated anemia is often considered within the broader spectrum of ACD, important differences exist between renal anemia and the inflammatory anemias seen in conditions such as rheumatoid arthritis, inflammatory bowel disease, and malignancy. In CKD, reduced erythropoietin production by the diseased kidney represents a major pathogenic mechanism, whereas in non-renal ACD, inflammation-driven iron sequestration and cytokine-mediated suppression of erythropoiesis play a more dominant role [6,8]. Nevertheless, substantial overlap exists between these entities, particularly through the central role of hepcidin, functional iron deficiency, and impaired erythropoietic responses. Furthermore, much of the available evidence regarding iron supplementation, erythropoiesis-stimulating agents, and emerging therapies originates from studies conducted in patients with CKD. For these reasons, the CKD-related literature has been included where it provides insight into shared pathophysiological mechanisms or therapeutic principles. However, findings from renal populations should not necessarily be extrapolated directly to other causes of ACD, and disease-specific evidence has been prioritized wherever available.

## 2. Methods

As this article was designed as a narrative review, a formal systematic review methodology was not employed. The relevant literature was identified through searches of PubMed, MEDLINE, and Google Scholar using combinations of the terms “anemia of chronic disease”, “anemia of inflammation”, “hepcidin”, “iron deficiency”, “erythropoiesis-stimulating agents”, and “functional iron deficiency”. Priority was given to contemporary reviews, international guidelines, landmark clinical studies, and publications with direct clinical relevance. Additional references were identified through a manual review of the cited literature.

## 3. Pathophysiology

**Figure 1:**
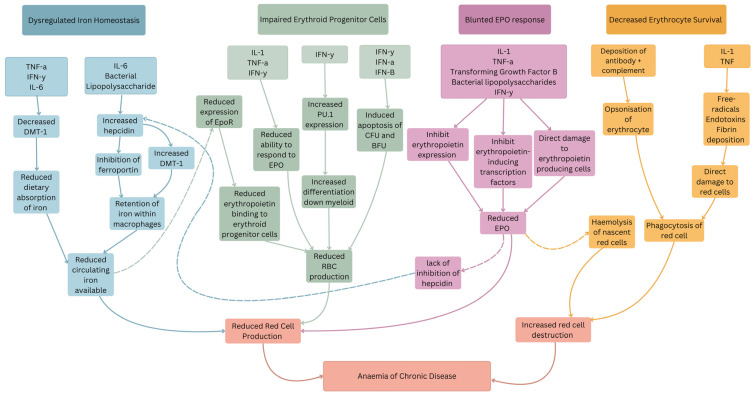
Pathophysiology of anemia of chronic disease.

Anemia of chronic disease develops through the interaction of chronic inflammation, dysregulated iron homeostasis, impaired erythropoiesis, inadequate erythropoietin (EPO) activity, and shortened erythrocyte survival. Although multiple cytokines contribute, IL-6-mediated induction of hepcidin is considered the central pathogenic mechanism [5,12]. These interconnected pathways are summarized in Figure 1, and the predominant inflammatory cytokines and their mechanisms are highlighted in Table 2.

**Dysregulated Iron Homeostasis**

The hallmark of ACD is functional iron deficiency, whereby total body iron stores are preserved or increased but become unavailable for erythropoiesis. This dysregulation of iron homeostasis is primarily driven by hepcidin, a protein produced mainly by hepatocytes in the liver, but also by adipocytes and macrophages [13]. Iron predominantly exists bound to hemoglobin, but small amounts are stored as ferritin in macrophages and hepatocytes and released into the circulation when iron levels are low [14]. Hepcidin inhibits the transmembrane ferroportin, so iron cannot efflux from macrophages and hepatocytes where it is stored [15,16]. Through this downregulation of ferroportin by hepcidin, there is a restricted availability of free iron for erythropoiesis [4]. Studies have identified that injecting mice with hepcidin can lead to prolonged low iron lasting up to 48 h [17]. However, the mechanism through which hepcidin binds and causes internalization of ferroportin is still largely unknown [17].

Under inflammatory conditions, Interleukin-6 (IL-6) upregulates hepcidin in a potent manner through the JAK2/STAT3 signaling pathway [18]. Bacterial lipopolysaccharide (LPS) is also thought to induce hepcidin through the BMP signaling pathway [19]. A study evaluating hepcidin’s role in inflammation showed that when human hepatocytes were stimulated with a panel of cytokines, IL-6 strongly induced the induction of hepcidin mRNA [19]. Additionally, inducing inflammation in mouse models led to increased hepcidin mRNA and decreased iron levels, but not in mice with hepcidin deficiency [20]. Another study showed that by infusing healthy volunteers with IL-6, the infusion resulted in a 7.5-fold increase in urinary hepcidin levels from baseline, and at the peak of hepcidin levels, serum iron had decreased by 34%, and serum transferrin saturation had decreased by 33% [12]. This gives evidence of both the role of IL-6 in hepcidin regulation and subsequently the impact of hepcidin on iron levels in the context of inflammation.

Divalent Metal Transporter 1 (DMT1) also plays a role in iron homeostasis, linked to hepcidin. Dietary iron is taken up in the small intestine via DMT1 on the apical membrane of enterocytes and is then transported to portal blood via ferroportin and stored [14,21]. Hepcidin has been shown to downregulate DMT-1 to reduce dietary iron uptake [22]. However, inflammatory mediators, including TNF-α, Interferon-γ (IFN-γ), and IL-6, have been shown to activate DMT-1, causing increased overall uptake and consequent retention of iron within macrophages via ferroportin inhibition [4].

Overall, these mechanisms lead to a functional iron deficiency, with a lack of iron available for erythropoiesis.

**Impairment of Erythroid Progenitor Cells**

Erythropoiesis is a result of the stepwise differentiation of cells from the multi-potent stem cell through the myeloid lineage, followed by commitment to the erythroid lineage, including the erythroblast and reticulocyte stages, to ultimately produce the red blood cell (RBC) [23]. As part of this process, the erythroid progenitor cells play a crucial role as the BFU-E (burst-forming unit-erythroid) is the first stage committed solely to the erythroid lineage and gives rise to the erythropoietin-sensitive CFU-E (colony-forming unit-erythroid) [24]. Therefore, impairment of these progenitor cells impacts red blood cell production.

Inflammatory mediators TNF, IL-1, IL-6, and IFN inhibit iron release from macrophages to the erythroid progenitors [4]. Iron levels that fall below a certain threshold trigger mechanisms in the erythroid progenitor cell that suppress its proliferation and differentiation. There is reduced expression of the erythropoietin receptor (EpoR), which is necessary for erythropoietin binding [25]. This EpoR expression is coordinated by Scribble, a regulator of receptor trafficking and signaling [26]. Reduced iron levels in the erythroid progenitor lead to scribble downregulation and therefore reduced EpoR expression [26].

As mentioned, erythropoietin (EPO) normally acts on erythroid progenitors to promote commitment to the erythroid lineage and ensure survival, proliferation, and differentiation of erythroblasts [26]. In chronic inflammation, IL-1, TNF-α, and INF-γ also reduce the ability of erythroid progenitors to respond to EPO [27]. In a study of patients with active rheumatoid arthritis, TNF-α was associated with a reduced number of erythroid progenitors [5]. IFN-γ appears to be the most potent inhibitor of erythroid burst-forming and erythroid colony-forming units, and there is an inverse correlation with Hb concentration/reticulocyte counts and levels of IFN-γ [3].

Further to the impairment of erythroid progenitor cells, IFN-γ is also thought to increase the expression of the PU.1 transcription factor, which promotes differentiation through the myeloid lineage from the common myeloid progenitor, thereby diverting from erythroid differentiation [23]. IFN-γ, IFN-α, and IFN-β additionally induce apoptosis of the erythroid burst-forming and colony-forming units through ceramide pathways or radical formation [27]. There is even some reduced expression of other growth factors, such as stem cell factor, due to IFN much earlier in the production process, contributing to overall decreased erythropoiesis [4].

Overall, the above highlights that, as well as the reduced availability of iron to these cells, inhibited response to EPO and apoptosis of the cells contribute to the diminished red blood cell count in chronic inflammation [19,20,21,22].

**Reduced EPO Production**

In healthy individuals, declining hemoglobin concentrations stimulate renal erythropoietin production. In ACD, this response is blunted relative to the severity of anemia [28]. IL-1, TNF-α, and TGF-β suppress erythropoietin synthesis through interference with transcriptional regulatory pathways and direct effects on renal EPO-producing cells [27,29]. Consequently, circulating erythropoietin levels are often inappropriately low for the degree of anemia.

**Decreased Erythrocyte Survival**

The lifespan of circulating erythrocytes is modestly shortened in ACD. Cytokine-mediated oxidative stress and mechanical damage, opsonization of erythrocytes, and increased erythrophagocytosis contribute to accelerated red cell clearance [4,30,31]. Cytokines IL-1 and TNF, and complement, are identified as having a role in this. Decreased EPO availability is also believed to cause rapid destruction of nascent red cells through hemolysis [27]. Although reduced erythrocyte survival is generally less important than iron restriction and impaired erythropoiesis, it contributes to the overall severity of anemia, particularly in patients with a significant inflammatory burden.

**Clinical Implications**

The pathogenesis of ACD is multifactorial; however, three mechanisms dominate: (1) hepcidin-mediated iron sequestration, (2) suppression of erythropoiesis, and (3) an inadequate erythropoietin response. Recognition of these pathways underpins current therapeutic approaches, including iron supplementation, erythropoiesis-stimulating agents, and emerging therapies targeting hepcidin and iron regulation. Ultimately, treating the underlying condition reduces the circulating inflammatory mediators responsible for driving ACD.

## 4. Diagnosis

As mentioned earlier, anemia is diagnosed based on low hemoglobin levels—defined as a Hb concentration <120 g/L in women and <130 g/L in men [30]. Those with ACD most typically show mild-to-moderate anemia, with Hb levels of 80–95 g/L, but levels can be lower [3,4]. Regardless of the underlying cause, anemia can present symptomatically with weakness, fatigue, reduced exercise tolerance, and difficulties with concentration and memory [1].

Diagnosis of anemia of chronic disease remains challenging because no universally accepted diagnostic criteria currently exist, and often it is considered a diagnosis of exclusion [6]. In practice, diagnosis relies upon the integration of clinical context, evidence of chronic inflammation, and laboratory findings consistent with iron-restricted erythropoiesis [30].

The most important diagnostic challenge is distinguishing isolated ACD from iron deficiency anemia (IDA) and mixed ACD/IDA. Although it may seem straightforward to diagnose patients with a known chronic inflammatory or malignant disorder with ACD when low Hb levels are seen, between 20% and 85% of patients with ACD also have true iron deficiency anemia. This may be disease-related or due to unrelated causes of iron-deficiency anemia, such as gastrointestinal (GI)/urogenital bleeding, frequent blood sampling, or losses from associated procedures, e.g., hemodialysis [30,32]. Laboratory findings in ACD, IDA, and mixed ACD/IDA are compared in Table 3. This distinction is clinically important because treatment strategies differ substantially. Patients with absolute iron deficiency require iron replacement and investigation for potential sources of blood loss, whereas management of isolated ACD is directed primarily toward treatment of the underlying inflammatory condition and optimization of erythropoiesis [33].

**Figure 2:**
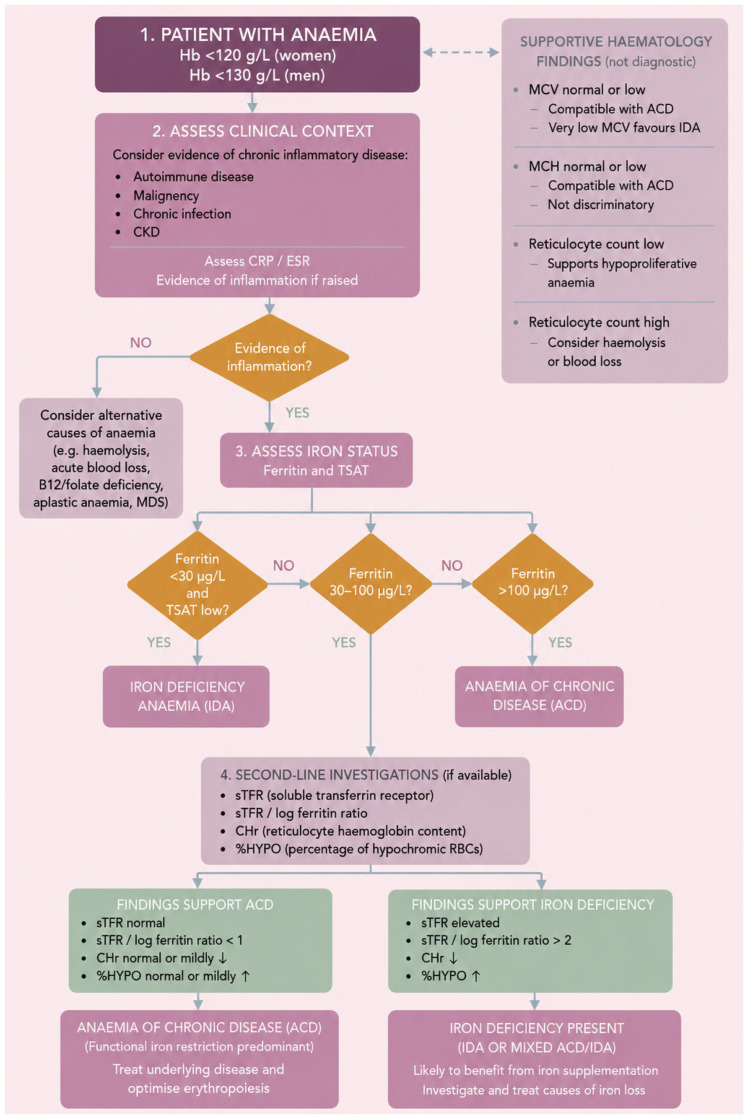
Diagnostic algorithm for anemia of chronic disease and mixed ACD.

A practical diagnostic approach to help identify ACD is proposed in Figure 2.

**First-Line Investigations**
**MCV and MCH**

Mean Corpuscular Volume (MCV) is a measure of the size of circulating red blood cells [34]. Anemia of chronic disease typically presents as a normocytic (normal MCV), normochromic anemia, but becomes microcytic with associated iron deficiency [23]. However, it is important to note that a normocytic anemia does not necessarily exclude iron deficiency, as it is possible to have a normocytic anemia as well as iron deficiency [23,35]. Mean corpuscular hemoglobin (MCH) is the most direct measure of the iron supply to the developing erythron, although a late reflection of it. An MCH <28 pg is suggestive of functional iron deficiency [1]. However, this is indistinguishable from iron-deficiency anemia. Therefore, while low MCH is supportive of ACD, it is of less value in differentiating ACD from IDA [36].

**Iron Studies**

An exploration of iron studies, a panel of tests used in the assessment of circulating and storage iron, forms a crucial part of the diagnosis of ACD and exclusion of IDA [37]. Serum iron levels, serum ferritin, transferrin levels, and transferrin saturations (TSAT) must all be taken into consideration.

Serum iron levels, measuring the amount of iron bound to transferrin in plasma as a reflection of available circulating iron, will be decreased both in ACD and IDA due to absolute iron deficiency in IDA and functional iron deficiency in ACD [37]. Since there is reduced circulating iron, there is less iron bound to transferrin, leading to reduced transferrin saturations also in both ACD and IDA [38]. Results begin to diverge when exploring the levels of the transferrin transporter, as these often increase in iron-deficiency anemia as a response to the low iron levels [39]. However, transferrin levels are sometimes decreased (or can also be normal) in ACD because cytokines negatively affect the expression of transferrin receptors [40]. As a result, the transferrin saturation decrease is more pronounced in IDA, as there is reduced iron availability for a proportionally greater number of receptors [3]. Although helpful in guiding diagnosis, transferrin and transferrin saturation levels alone lack the sensitivity and specificity to definitively diagnose anemia of chronic disease [9,41,42].

**Ferritin**

Ferritin is an acute-phase protein and therefore levels increase in inflammation irrespective of iron stores [43]. Levels are also thought to rise in ACD due to increased iron retention within macrophages [44]. Serum ferritin is widely considered the most effective non-invasive test for differentiating IDA from ACD [8,45].

A ferritin concentration <30 μg/L strongly suggests absolute iron deficiency, whereas values >100 μg/L generally favor ACD [30]. However, normal or elevated ferritin levels do not exclude iron deficiency in the presence of inflammation. Patients with ferritin concentrations between 30 and 100 μg/L represent a diagnostic grey zone in whom coexisting IDA should be considered, particularly if transferrin saturation is reduced [33,46]. Galloway and Smellie suggested that in patients with chronic inflammation or liver disease, iron deficiency remains likely until ferritin concentrations exceed approximately 70 μg/L, highlighting the need for cautious interpretation in these settings [35]. In essence, low ferritin is highly informative; normal ferritin is not.

Ferritin may be particularly helpful in predicting response to iron therapy rather than confirming a diagnosis of ACD. Current guidelines acknowledge that functional iron deficiency can exist despite elevated ferritin concentrations, especially in CKD, where patients may respond to intravenous iron despite ferritin levels well above the normal range [6,46]. Ferritin should therefore be interpreted alongside markers of iron availability, such as transferrin saturation, and within the broader clinical context.

**Reticulocyte Count**

The reticulocyte count gives information on the productivity of the erythroid marrow, and the reticulocyte percentage can be an inverse measure of red cell lifespan [1]. A low reticulocyte count is seen in anemia of chronic disease due to its hypoproliferative nature, with reduced iron availability and response to EPO, causing underproduction of red cells [16]. Reticulocyte count will be reduced in IDA as well as ACD, so it is not helpful in distinguishing between the two [4].

**Inflammatory Markers**

Essential to a diagnosis of anemia of chronic disease is the consideration of the chronic inflammation that leads to abnormal red cell production. This can be inferred through the detection of non-specific inflammatory markers such as raised C-Reactive Protein (CRP) and Erythrocyte Sedimentation Rate (ESR), as well as thrombocytosis and leukocytosis [33]. In patients with rheumatoid arthritis and anemia of chronic disease, iron absorption was found to be inversely correlated with ESR and CRP [47].

**Second-Line Investigations**
**sTfR, and sTfR/log Ferritin Ratio**

Serum transferrin receptor (sTfR) is a fragment of the transferrin membrane receptor and, in response to low iron availability for erythropoiesis, the expression of sTfR is increased [48]. Comparatively, its expression is reduced by inflammatory cytokines, and assays of sTfR are typically normal in anemia of chronic disease [48]. The main limitation of this investigation is that the assay is expensive and not standardized or widely available in current practice [6]. In a retrospective analysis on the use of erythropoietin to treat ACD, there was found to be no evidence for the usefulness of sTfR in predicting iron-deficient erythropoiesis or monitoring the correction of ACD [49]. Instead, the ratio of sTfR to the log of ferritin levels, also termed the ferritin index, is proposed to be helpful in distinguishing ACD from IDA [41]. A ratio of <1 is consistent with anemia of chronic disease, and a ratio >2 suggests iron deficiency anemia [50]. However, as with sTfR, this index calculator is not widely used in current clinical practice. Therefore, the sTfR/log ferritin ratio is a very useful tool, but requires more widespread availability and still needs to be interpreted alongside other markers and clinical context.

**CHr and %HYPO**

Reticulocyte hemoglobin content (CHr) and percentage hypochromic red cells (%HYPO) are markers of iron-restricted erythropoiesis that provide information on iron availability to developing erythrocytes [48,51]. CHr reflects hemoglobin incorporation into newly produced red cells over the preceding 48 h and may identify iron deficiency earlier than conventional indices [49]. Low CHr values are associated with iron deficiency and may help distinguish isolated ACD from mixed ACD/IDA, although reported diagnostic thresholds vary between studies [48,50].

By contrast, %HYPO reflects the proportion of mature red cells with reduced hemoglobin content and provides a longer-term and more sensitive assessment of iron-restricted erythropoiesis [51,52]. %HYPO values > 10% are considered a direct indicator of functional iron deficiency and are incorporated into some renal and hematology guidelines, particularly in patients receiving erythropoiesis-stimulating agents [43,53].

Although both CHr and %HYPO may improve assessment of iron availability and guide decisions regarding iron supplementation, particularly in CKD, neither has been established as a definitive diagnostic test for ACD. Their use remains largely complementary to conventional iron studies, and wider adoption has been limited by issues relating to assay availability, standardization, and diagnostic thresholds [46,48].

**Emerging Biomarkers**
**Hepcidin**

Given the extent of the involvement of raised hepcidin levels in the pathophysiology of anemia of chronic disease, it is unsurprisingly given consideration as a diagnostic tool, being measured in urine or serum [23]. Although hepcidin levels are increased in ACD, a wide variety of inflammatory conditions non-specifically cause hepcidin rise, including rheumatological conditions, inflammatory bowel disease (IBD), lymphoma, myeloma, and critical illness [8]. In dialysis patients, hepcidin levels are increased due to the impaired hepcidin excretion and provide minimal value in diagnosis [53]. Importantly, concomitant iron deficiency acts in opposition to the hepcidin increase from inflammation, and therefore, hepcidin may be normal or low in these patients [51]. Similarly, in IBD, blood loss and malnutrition leading to iron deficiency can offset the hepcidin increase caused by inflammation [52]. Therefore, hepcidin use is also limited by the lack of an established assay, the limited number and quality of studies, and the variety of factors that impact hepcidin levels [52]. At this moment in time, hepcidin is only of academic interest and has no use in current practice.

**GDF15**

Growth Differentiation Factor 15 (GDF15) is an anti-inflammatory cytokine involved in the regulation of hepcidin [11]. Although several studies have reported elevated GDF15 concentrations in patients with anemia of chronic disease, findings remain inconsistent across different patient populations [7,11,54,55]. While some investigations have suggested a relationship between GDF15, iron metabolism, and inflammatory activity, others have failed to demonstrate a meaningful association with functional iron deficiency [8,54]. Consequently, the current evidence does not support the routine clinical use of GDF15 in the diagnosis or management of anemia of chronic disease. At present, GDF15 should be regarded as an investigational biomarker requiring further validation before incorporation into diagnostic algorithms.

**Erythropoietin**

Erythropoietin is produced by renal medullary cells in hypoxic and anemic states [56]. Although EPO concentrations are generally increased in patients with ACD compared with healthy controls, studies consistently demonstrate that this rise is inappropriately low relative to the severity of anemia. In particular, patients with ACD typically exhibit lower EPO levels than patients with iron deficiency anemia who have comparable hemoglobin concentrations [54,56,57]. This supports the concept of a relative erythropoietin deficiency or “blunted EPO response” in ACD.

While this finding contributes to the understanding of ACD pathophysiology and provides a rationale for the use of erythropoiesis-stimulating agents, measurement of serum EPO has limited diagnostic utility. No widely accepted diagnostic thresholds have been established, and current guidance does not recommend routine EPO measurement in the diagnostic evaluation of ACD [6,58].

**Other Investigations**
**Blood Film**

The blood film can provide valuable information on the underlying cause of ACD. This includes findings such as toxic granules in neutrophils seen in sepsis, thrombocytosis in chronic hemorrhage, mixed nutritional deficiency causing hypersegmented neutrophils, or folate/B12 deficiency seen in malignant conditions [4]. However, it still does not play a diagnostic role in identifying ACD.

**Bone Marrow Biopsy**

Bone marrow aspirate with Perl’s stain is considered the gold standard assessment of iron stores and may be helpful in excluding other causes of anemia, such as myelodysplastic syndromes [33]. Increased macrophage iron can be identified through Perl’s stain, which indicates abnormal bone marrow iron distribution, in keeping with the sequestration of iron to macrophages seen in ACD [59]. Most usefully, iron stores can be assessed to show a normal or increased iron store in ACD and a decreased iron store in mixed ACD/IDA [60].

When performed, a bone marrow biopsy in anemia of chronic disease shows a number of non-specific findings. Marrow can be hypo-, normo-, or hypercellular [60]. Hypocellularity is frequently seen in patients with kidney dysfunction, and more so in those with ESKD (End-Stage Kidney Disease) than with CKD [61]. Usually, there is erythroid hypoplasia, although there can be normal or increased erythropoiesis [60]. Most often, myeloid and megakaryocytic lines are increased, but these can also be normal or decreased [60]. Most cases show dysplasia in all cell lineages, but there are conflicting reports in the literature on whether this correlates with disease activity [60]. It is also possible to visualize iron-depleted erythroblasts and other non-specific inflammatory changes through more detailed bone marrow examination [59]. Despite being the gold standard for iron store evaluation, bone marrow biopsy is rarely recommended for the diagnosis of anemia of chronic disease due to its invasive nature and minimal value in providing a definitive diagnosis of ACD [6]. Interestingly, in a study of patients with rheumatoid arthritis, it was concluded that a combination of MCV, ferritin, and transferrin resulted in 100% validity in the identification of iron deficiency anemia when assessed against bone marrow biopsy [47]. Therefore, the interpretation of other serum parameters within the clinical context is much preferred to the use of bone marrow biopsy in the diagnosis of ACD.

**Figure 3:**
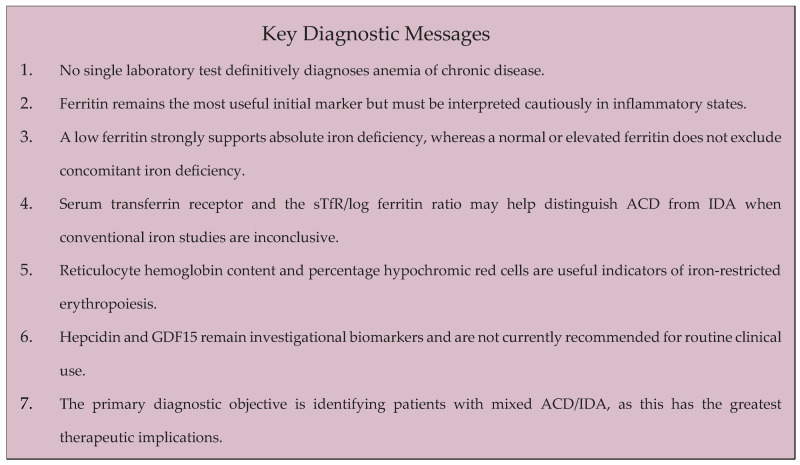
Key diagnostic messages.

A summary of the key diagnostic messages can be seen in Figure 3.

## 5. Management

**Should We Manage Anemia of Chronic Disease?**

The first key question in the management of ACD is whether there is a clinical benefit to managing the anemia in affected patients. Historically, anemia of chronic disease was considered a protective adaptation to inflammation, with iron sequestration limiting microbial proliferation and reducing iron availability to malignant cells [4,62]. Therefore, there have been some concerns that treatment of ACD could impact the underlying disease, with studies in environments with a high endemic burden of infectious disease showing mild anemia or iron deficiency to be protective against enteric infections and malaria [63,64,65].

However, accumulating evidence demonstrates that anemia is independently associated with poorer clinical outcomes across a wide range of chronic diseases [66]. It is independently associated with an increased risk of death in patients with renal impairment on dialysis, a poor prognostic indicator in cancer, as well as increased morbidity and mortality in patients with congestive heart failure and human immunodeficiency virus (HIV) [1,4].

Beyond physiological consequences, anemia contributes significantly to fatigue, exercise intolerance, cognitive dysfunction, and reduced quality of life. Patient-reported outcome studies consistently identify fatigue as one of the most burdensome symptoms experienced by individuals with chronic inflammatory diseases such as arthritis and cancer [1,46]. Consequently, contemporary management strategies aim not only to improve laboratory parameters but also to alleviate symptoms and improve overall patient well-being [1].

Treatment decisions should therefore balance potential benefits against disease-specific risks, recognizing that correction of anemia may not always be appropriate in every clinical context.

**Treat Underlying Cause**

It is undisputed in the literature that the most effective treatment for ACD is treatment of the underlying condition, which is associated with resolution of anemia [3,5,30]. This is seen in the use of TNF-α inhibitors for the treatment of rheumatoid arthritis or inflammatory bowel disease, anti-retroviral therapy in HIV infection, and corticosteroids in polymyalgia rheumatica [67,68].

The main limitation of this approach is that it is not always possible. Patients with chronic kidney disease or heart failure not suitable for transplant, or patients with incurable cancer, cannot look to these options for resolution of anemia [5,8].

**Erythropoietin-Stimulating Agents**

Erythropoiesis-stimulating agents (ESAs) are recombinant forms of human erythropoietin that address one of the key pathophysiological features of anemia of chronic disease (ACD): an inadequate erythropoietin response relative to the severity of anemia [69]. By stimulating erythroid progenitor proliferation and differentiation, ESAs can partially overcome inflammation-mediated suppression of erythropoiesis and improve hemoglobin (Hb) concentrations, particularly when combined with iron supplementation [70]. Currently available ESAs in the UK include epoetin alfa, epoetin beta, epoetin theta, epoetin zeta, and darbepoetin alfa, with licensed indications primarily in chronic kidney disease (CKD) and chemotherapy-associated anemia [58,71].

The clinical benefit of ESAs varies substantially according to the underlying disease process. The strongest evidence exists in CKD and chemotherapy-associated anemia, where ESA therapy consistently increases Hb levels, reduces transfusion requirements, and improves patient-reported quality of life [1,6,72,73,74]. In CKD, ESA treatment forms a cornerstone of anemia management because endogenous erythropoietin deficiency contributes directly to disease pathogenesis [75,76]. Similarly, in patients receiving chemotherapy, ESAs may reduce transfusion exposure and improve symptoms when used in carefully selected patients after correction of iron deficiency and other reversible causes of anemia [74,77].

Evidence supporting ESA use in other inflammatory disorders is less robust. Small studies in rheumatoid arthritis and inflammatory bowel disease have demonstrated improvements in hemoglobin concentration, particularly when ESAs are combined with iron therapy [78,79]. However, the emergence of effective disease-modifying therapies, including biologic agents and JAK1 inhibitors, has shifted treatment priorities towards control of the underlying inflammatory process, which itself improves anemia [67,80,81]. Similarly, although ESAs have historically been used in HIV-associated anemia, subsequent systematic review evidence has failed to demonstrate consistent improvements in hemoglobin, transfusion requirements, quality of life, or survival, limiting their contemporary role in this setting [82,83,84,85,86,87].

The benefits of ESA therapy must be balanced against well-recognized safety concerns. Across multiple disease settings, ESA treatment reduces transfusion requirements and can improve quality of life, but these benefits are accompanied by increased risks of thromboembolic and cardiovascular events, particularly when Hb concentrations exceed 120 g/L [77,88]. Consequently, contemporary guidelines recommend using the lowest effective ESA dose and avoiding normalization of hemoglobin levels [8].

Particular concern has surrounded the use in oncology. Early randomized trials reported worse oncological outcomes among some patients receiving ESAs. In head and neck cancer, Henke et al. demonstrated poorer locoregional tumor control and reduced progression-free survival among patients receiving epoetin beta during radiotherapy [89]. Similarly, Leyland-Jones et al. reported increased mortality in patients with metastatic breast cancer treated with epoetin alfa [90]. These findings prompted regulatory warnings from the US Food and Drug Administration (FDA) and contributed to more restrictive prescribing recommendations [33]. However, subsequent meta-analyses have produced conflicting results, with several failing to demonstrate a consistent increase in tumor progression or overall mortality, while continuing to confirm an increased risk of venous thromboembolism [91,92,93,94]. The overall balance of evidence therefore supports ESA use only in carefully selected cancer patients, particularly those receiving myelosuppressive chemotherapy, where reductions in transfusion requirements may outweigh potential risks. Key studies highlighting safety considerations in ESAs are highlighted in Table 4.

Current guidance reflects this risk-benefit framework and is summarized in Table 5. Although recommendations vary between organizations, there is broad agreement that ESAs should be reserved for symptomatic patients with clearly defined indications, initiated only after correction of iron deficiency, and used to achieve modest Hb targets rather than complete normalization [77]. Consequently, ESA therapy remains standard practice in CKD and selected patients with chemotherapy-associated anemia, whereas its role in other forms of ACD is more limited and should be considered on an individual basis. Overall, ESAs are best viewed as targeted therapies for selected patient populations rather than a universal treatment for ACD.

**Iron Replacement**

Iron supplementation remains an important component of anemia management in patients with chronic inflammatory disorders, although its role differs according to whether iron deficiency is absolute or functional, and to what degree.

Oral iron supplementation is inexpensive, widely available, and frequently used as first-line treatment when iron deficiency is suspected [8]. However, elevated hepcidin concentrations in inflammatory states substantially reduce gastrointestinal iron absorption, limiting its efficacy in many patients with ACD [6]. For this reason, oral iron is most effective in patients with coexisting absolute iron deficiency and relatively low levels of inflammation, where outcomes may be comparable to intravenous (IV) preparations [96]. The principal rationale for oral iron use in ACD, therefore, relates less to treatment of the inflammatory anemia itself and more to correction of the iron deficiency that commonly accompanies it.

Intravenous iron has attracted greater interest in ACD because it bypasses hepcidin-dependent intestinal absorption and can rapidly increase iron availability for erythropoiesis [6,97]. Clinical studies have demonstrated improvements in hemoglobin levels across several chronic inflammatory conditions, including inflammatory bowel disease (IBD), rheumatoid arthritis, and selected malignancies [68,98,99,100,101]. However, interpretation of these studies is complicated by the frequent coexistence of absolute iron deficiency, making it difficult to determine the extent to which observed benefits reflect correction of iron deficiency rather than treatment of ACD itself. Consequently, while IV iron is clearly effective in patients with combined ACD and iron deficiency, evidence supporting its use as monotherapy in pure ACD remains comparatively limited [8,102].

The strongest evidence for iron therapy in ACD exists when it is combined with erythropoiesis-stimulating agents (ESAs). Iron-restricted erythropoiesis commonly develops during ESA treatment as iron demand exceeds the capacity of reticuloendothelial iron stores to supply the bone marrow [97]. In this setting, IV iron improves hemoglobin responses, reduces ESA dose requirements, and may enhance quality of life [58,97,101]. Benefits have been demonstrated in several disease settings, although much of the evidence originates from populations in whom iron deficiency frequently coexists [49,101]. The available literature therefore supports IV iron as an adjunct to ESA therapy, particularly when laboratory findings suggest functional iron deficiency or an inadequate response to ESAs alone.

Potential risks of iron supplementation must also be considered. Concerns have been raised from observational studies reporting associations between IV iron and increased infection risk, stating that increased circulating iron promotes pathogen growth [103]. However, more recent clinical studies have not consistently demonstrated worsening of infection or adverse outcomes following iron administration, but instead reported improved hemoglobin recovery and higher survival without evidence of infection progression [104]. Current evidence therefore suggests that theoretical risks should be balanced against the well-established benefits of correcting iron-restricted erythropoiesis, particularly in symptomatic patients.

Overall, iron supplementation should be considered in patients with ACD who have coexisting absolute iron deficiency, laboratory evidence of functional iron deficiency, or an inadequate response to ESA therapy. Intravenous iron is generally preferred when inflammation is significant, oral iron is poorly tolerated or ineffective, or to maintain iron stores throughout ESA therapy. In contrast, routine iron administration in patients with ACD who have adequate iron stores remains insufficiently supported by current evidence. Management should therefore be guided by a careful assessment of iron status, including ferritin and transferrin saturation, to identify those most likely to benefit while avoiding unnecessary iron exposure.

**Hypoxia-Inducible Factor Prolyl Hydroxylase Inhibitors**

An emerging treatment for anemia of chronic disease has arisen in the form of Hypoxia-Inducible Factor Prolyl Hydroxylase Inhibitors (HIF-PHIs). These have initially been trialed for the oral treatment of anemia in CKD. HIF-PHIs stimulate exposure to moderate hypoxia, which triggers the production of erythropoietin and the delivery of iron from enterocytes and macrophages [105]. One study compared the efficacy and safety of HIF-PHIs with ESAs in anemic CKD patients not on dialysis. The results of the study showed an equal increase in Hb across all ESAs and HIF-PHIs, with the exception of a smaller increase with the use of the PHI vadadustat. There was also no difference in all-cause mortality [106]. Other trials have also shown vadadustat to be equally effective in other HIF-PHIs, compared to ESAs and placebo, but with a slightly higher cardiovascular/thrombotic risk [105]. HIF-PHIs have been included by the UK Kidney Association in the most recent guidance, which recommends offering a HIF-PHI agent after iron repletion to patients with symptomatic anemias or who are intolerant to ESAs [58]. HIF-PHIs are a promising novel treatment that now form part of the management of anemia of CKD, but require further research, including trials on their use in other inflammatory conditions associated with anemia of chronic disease.

**Transfusion**

It is widely agreed in the literature that transfusion is not an effective or appropriate management option for anemia of chronic disease [3,30,33]. Transfusion has its place in the management of severe or life-threatening anemia, when rapid correction of anemia is required to clinically stabilize the patient. However, the risks associated with transfusion, including iron overload, sensitization to human leukocyte antigens (HLA) (particularly in CKD patients considering renal transplant), possible viral transmission, and the expense of the resource, are all considered not to outweigh the benefits [107]. To further emphasize this, clinical trials of cancer patients on chemotherapy who had anemia treated with blood transfusions were shown to have a worse quality of life than those managed with ESAs [1].

**Hepcidin Inhibitors + Anti-Hepcidin Antibodies**

Given the key involvement of hepcidin in the pathophysiology of anemia of chronic disease, antagonists to the action of hepcidin are given consideration in its management [8]. High levels of hepcidin have been associated with a poor response to ESAs, suggesting there may be a place for anti-hepcidin drugs in the form of anti-hepcidin antibodies or hepcidin inhibitors such as NOX-H94 [4].

Most studies evaluating hepcidin-targeted therapies have been conducted in preclinical models, with a particular focus on the BMP pathway, which is known to induce hepcidin. Soluble hemojuvelin or dorsomorphin both block hepcidin production by inhibiting BMP signaling and reverse anemia of inflammation in rat models [108]. Dorsomorphin has additionally demonstrated in vivo inhibition of both BMP and IL-6 signaling pathways in zebrafish models [109]. Similarly, soluble hemojuvelin has been shown to reduce hepcidin expression and increase circulating iron levels in murine models of inflammatory bowel disease [110].

Other agents targeting the BMP pathway have also demonstrated potential therapeutic benefit. Heparin can bind BMP proteins and disrupt downstream signaling, resulting in reduced hepatic hepcidin expression and increased serum iron levels in animal models [111]. In addition, therapies that modulate the BMP pathway, such as luspatercept (a transforming growth factor-β ligand trap) and momelotinib (a JAK2/activin receptor-like kinase 2 inhibitor), are already approved for the treatment of anemia associated with myelodysplastic syndromes and myelofibrosis, respectively [112].

Anti-IL6 (Tocilizumab) is one receptor antibody used to decrease hepcidin production and was found to reverse inflammation-associated anemia in monkeys [113]. It has also been trialed in patients with the multicentric form of Castleman disease, a lymphoproliferative disorder, showing reductions in serum hepcidin and improvement in anemia [114]. Other trials with anti-hepcidin antibodies showed decreased hepcidin without significant Hb increase, but increased sensitivity to EPO treatment, leading to resolution of anemia [115]. Antibodies targeting the binding of hepcidin to ferroportin have also been demonstrated to raise serum iron in monkeys [17].

Despite encouraging preclinical and early clinical findings, hepcidin-targeted therapies have not yet been established as a standard treatment for ACD. Current evidence remains limited, with most data derived from animal studies or early-phase clinical trials. While combination therapy with hepcidin inhibitors and ESAs may represent a promising strategy by improving iron availability and reducing ESA requirements, supporting evidence is largely restricted to experimental models [109,116]. Consequently, hepcidin-directed therapies should currently be regarded as investigational, and further randomized clinical trials are required before their routine use can be recommended.

**Figure 4:**
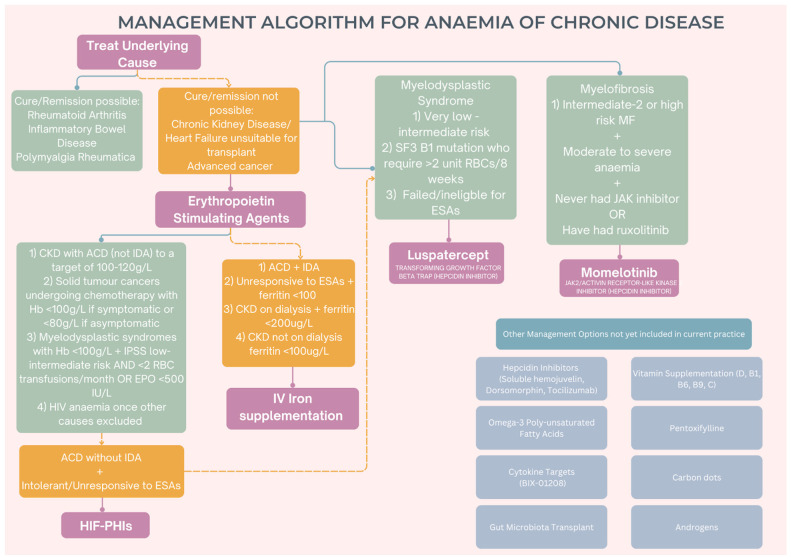
Management algorithm for anemia of chronic disease.

A flowchart summarizing the management of ACD is included in Figure 4.

**Other Novel Therapies**

Several other novel therapies have been proposed to have utility in the management of ACD, including omega-3 fatty acids, cytokine targets, gut microbiota transplant, pentoxifylline, vitamin supplementation, carbon dots, and androgens. These novel therapies have been included to provide a comprehensive synthesis of existing guidelines alongside emerging therapeutic avenues, but must be clearly distinguished as highly experimental at present and separate from the current standard of care.

**Omega-3 Polyunsaturated Fatty Acids**

Omega-3 polyunsaturated fatty acids are a novel therapy under consideration for the management of ACD. Some observational studies have shown a downregulation of TNF-α and IL-6, and potential benefits in the treatment of rheumatoid arthritis and diabetes [4]. However, data is still conflicting, with other studies reporting no statistically significant difference in inflammatory markers and no benefits to anemia in patients treated with omega-3 fatty acids compared to placebo in patients on hemodialysis [117].

**Cytokine Targets**

The p38 mitogen-activated protein kinase (MAPK) pathway is known to be activated by a number of cytokines, including IFNs, TNF-α, and TGFB, and exerts myelosuppressive effects. Studies have suggested that inhibiting p38 using BIX-01208 can reverse these myelosuppressive effects and partially reverse the suppression of erythropoiesis seen in patients with myelodysplastic syndrome or anemia of chronic disease [118].

**Gut Microbiota Transplant**

The gut microbiome has attracted significant interest in recent years for its interplay with a range of diseases, with some evidence to suggest that there is also a role in hematopoiesis. Fecal microbial profiles are altered in patients with ACD, and one study showed fecal microbiota transplantation from a healthy donor into patients with ACD significantly increased hemoglobin. The suggestion is that certain species of bacteria associated with reduced inflammation and higher hemoglobin levels are considerably less abundant in patients with ACD compared to healthy controls [119].

**Vitamin Supplementation**

Studies have identified an association between vitamin D deficiency and anemia of chronic disease in elderly populations, with anemic individuals being twice as likely to have vitamin D deficiency compared to non-anemic individuals [120]. However, there has been little research to explore the pathophysiology behind this or the therapeutic benefit of vitamin D supplementation [8].

The supplementation of essential amino acids has also been given consideration in the treatment of ACD, in view of the production of A-amino-levulinic acid being the rate-limiting step in the synthesis of the heme ring. One study reviewing the treatment of anemia of chronic disease in heart failure patients with iron therapy and supplementation of essential amino acids and vitamins B1, B6, B9, and D found that there was a significantly faster increase in hemoglobin compared to patients on standard iron therapy [66].

Vitamin C is another vitamin theorized to have a role in the management of ACD. It is known to downregulate hepcidin expression in liver cells and has been shown to improve hemoglobin when given alongside iron supplementation in patients with iron-refractory iron deficiency anemia, whose underlying genetic mutation affects similar pathways to anemia of chronic disease. There have not been any clinical trials to explore this further [121].

**Pentoxifylline**

Pentoxifylline is another novel agent with studies showing its suppressive effects on TNF-α and IFN-γ. There have been trials showing that the use of pentoxifylline in CKD patients with anemia not responsive to EPO can improve hemoglobin levels [122].

**Carbon Dots**

Carbon dots have been hypothesized as a potential therapeutic agent in the treatment of anemia. Carbon dots are carbon-based nano-materials that stimulate self-renewal of erythroid progenitor cells and have been shown to promote erythrocyte production in vitro and generate significant increases in red cell indices of mice when injected with carbon dots. They have been suggested as a potential therapeutic agent for cancer-related anemia to try to avoid the concerns raised regarding the adverse effects of ESAs [123].

**Androgens**

Androgens are known to downregulate hepatic hepcidin messenger RNA via the BMP signaling pathway, and also upregulate EPO mRNA expression, so they may have a future role in the management of anemia of chronic disease. At present, the evidence regarding the use of synthetic androgen Danazol has shown it is effective in treating anemia related to myelofibrosis, with one trial identifying a stabilization of hemoglobin in 55% of patients [124]. However, monitoring of liver function tests and prostate-specific antigen is recommended with its use due to the increased risk of deranged liver function and prostate cancer, respectively [125]. Little work has been performed to evaluate their wider use in ACD [121].

## 6. Conclusions

To conclude, this narrative review offers an update on the pathophysiology of anemia of chronic disease and recent treatments. Anemia of chronic disease is driven by several inflammatory mediators, primarily IL-6, which lead to dysregulated iron homeostasis, creating a functional iron deficiency state, inhibition of erythropoietin and erythroid progenitor cells, and decreased erythrocyte survival. Serum markers, including iron studies, ferritin, transferrin receptors, and reticulocyte hemoglobin content, are valuable in building a picture of anemia of chronic disease and identifying or excluding co-existent iron deficiency. The utility of markers such as erythropoietin, GDF15, and hepcidin has also been discussed. Current management guidelines involve treatment of the underlying disease, in addition to the use of erythropoietin-stimulating agents +/− iron supplementation and consideration of HIF-PHIs and hepcidin inhibitors in specific circumstances. Other novel treatments are emerging. Overall, it is clear that much work is still needed to improve our understanding and management of this complex condition.

## Figures and Tables

**Table 1:** Diseases associated with anemia of chronic disease.

Category	Primary Underlying Cause
Infection (acute and chronic)	Bacterial (Tuberculosis, Osteomyelitis) Fungal Viral (Human Immunodeficiency Virus, Hepatitis) Parasitic (Malaria)
Cancer	Hematological (myeloma, lymphoma, myeloproliferative disorders) Solid Tumors
Autoimmune disease	Rheumatoid arthritis Systemic Lupus Erythematosus Inflammatory bowel disease Sarcoidosis Vasculitis
Chronic Organ Failure	Heart Failure Chronic Kidney Disease Chronic Obstructive Pulmonary Disease
Organ Transplant Rejection	
Ageing	

**Table 2:** Key inflammatory mediators implicated in anemia of chronic disease.

Inflammatory Mediator	Principal Mechanisms Contributing to Anemia
IL-6	Upregulates hepcidin via JAK/STAT signaling, reducing iron availability
TNF-α	Suppresses erythropoiesis, impairs EPO production, and reduces erythrocyte survival
IL-1	Suppresses EPO production and responsiveness
IFN-γ	Inhibits erythroid progenitor proliferation and promotes apoptosis
TGF-β	Suppresses renal EPO production
Hepcidin	Causes iron sequestration through ferroportin degradation

**Table 3:** Comparison of parameters in the assessment of ACD vs IDA vs ACD/IDA.

Parameter	ACD	IDA	ACD/IDA
Hemoglobin	↓	↓	↓
MCV	Normal or ↓	Normal to ↓↓↓	Normal or ↓
MCH	Normal or ↓	↓	↓
Serum iron	↓	↓	↓
Transferrin	Normal or ↓	Normal or ↑	Variable
Transferrin saturation	↓	↓↓	↓↓
Ferritin	Normal or ↑	↓	Variable (often normal or mildly reduced)
Reticulocyte count	↓	↓	↓
CRP/ESR	↑	Normal	↑
sTfR	Normal	↑	↑
sTfR/log ferritin ratio	<1	>2	Intermediate
CHr	Normal or mildly ↓	↓	↓
%HYPO	Normal or ↑	↑	↑
Hepcidin	↑	↓	Variable
GDF15	Variable ↑	Normal	Variable ↑
EPO	Inappropriately normal or ↓	↑	Variable
Bone marrow iron stores	Normal or	↓	↓

↓; Decreased, ↑; Increased; MCV; mean corpuscular volume, MCH; mean corpuscular haemoglobin, CRP; C-reactive protein, ESR; Erythrocyte sedimentation rate, sTfR; Soluble transferrin receptor, CHr; Reticulocyte haemoglobin content, %HYPO; Percentage of hypochromic red blood cells, GDF15; Growth differentiation factor 15.

**Table 4:** Safety considerations in trials of ESAs.

Trial/Study	Population	Principal Finding	Safety Concern
Henke et al., 2003 [89]	Head and neck cancer	Improved Hb	Reduced locoregional control and survival
Leyland-Jones et al., 2003 [90]	Metastatic breast cancer	Study terminated early	Increased mortality due to disease progression and thrombotic events
Singh et al., 2006 [88]	CKD	Higher Hb target achieved	Increased cardiovascular events
Pfeffer et al., 2009 [95]	CKD and diabetes	Reduced transfusions	Increased stroke risk
Vansteenkiste et al., 2012 [92]	Lung cancer	Improved Hb Reduced transfusion requirements No significant adverse effects on overall survival or disease progression	Increased risk of thromboembolic events
Tong et al., 2024 [93]	Lung cancer	Reduced transfusion requirements No statistically significant increase in mortality	Higher incidence of thrombotic vascular events
Glaspy et al., 2010 [94]	All cancer types	No significant increase in mortality or disease progression overall	Increased risk of venous thromboembolism

**Table 5:** Comparison of major ESA guideline recommendations.

Guideline	Indication	Hb Threshold for Initiation	Target Hb
National Institute for Clinical Excellence (UK) [71]	People with cancer who are undergoing chemotherapy	Not specified	Not specified
European Society of Medical Oncology [77]	Solid tumor cancer patients receiving chemotherapy/chemoradiotherapy	<80 g/L if asymptomatic, <100 g/L if symptomatic	Avoid >120 g/L
	Low/intermediate risk MDS with EPO <500 IU/L and/or <2 RBC transfusions per month	<100 g/L	Individualized
European Organization for Research and Treatment of Cancer [91]	Cancer patients receiving chemotherapy/radiotherapy	90–110 g/L, depending on symptoms	120–130 g/L
	Cancer patients not receiving chemotherapy/radiotherapy		
UK Kidney Association [58]	Anemia of chronic disease, once pure iron deficiency excluded, and patients are iron replete	<100 g/L	100–120 g/L

## Data Availability

No new data were created or analyzed in this study.

## Abbreviations

The following abbreviations are used in this manuscript:

ACD	Anemia of chronic disease
BCSH	British Committee for Standards in Hematology
BFU-E	Burst Forming Unit Erythroid
CFU-E	Colony Forming Unit Erythroid
CHr	Reticulocyte hemoglobin content
CKD	Chronic Kidney Disease
CRP	C-Reactive Protein
DMT1	Divalent metal transporter 1
EORTC	European Organization for Research and Treatment of Cancer
EPO	Erythropoietin
ESMO	European Society of Medical Oncology
ESA	Erythropoietin-stimulating agents
ESKD	End-Stage Kidney Disease
ESR	Erythrocyte Sedimentation Rate
FDA	Food and Drug Administration
GDF15	Growth Differentiation Factor 15
Hb	Hemoglobin
HIF-PHIs	Hypoxia-Inducible Factor Prolyl Hydroxylase Inhibitors
HIV	Human Immunodeficiency Virus
HLA	Human Leukocyte Antigens
IBD	Inflammatory Bowel Disease
IDA	Iron deficiency anemia
IFN	Interferon
IL	Interleukin
IPSS	International Prognostic Scoring System
IV	Intravenous
LPS	Lipopolysaccharide
MAPK	Mitogen-Activated Protein Kinase
MCH	Mean Corpuscular Hemoglobin
MCV	Mean Corpuscular Volume
MDS	Myelodysplastic Syndrome
NICE	National Institute for Health and Care Excellence
RBC	Red blood cell
sTfR	Serum Transferrin Receptor
TNF	Tumor necrosis factor
TSAT	Transferrin Saturation
%HYPO	% of hypochromic red cells
